# Comparative single-dose pharmacokinetics of sildenafil after oral and rectal administration in healthy beagle dogs

**DOI:** 10.1186/s12917-018-1617-7

**Published:** 2018-09-24

**Authors:** Hyuck-Joo Yang, Ye-In Oh, Jong-Woo Jeong, Kun-Ho Song, Tae-Sung Koo, Kyoung-Won Seo

**Affiliations:** 1 Department of Veterinary Internal Medicine, College of Veterinary Medicine, Chungnam National University, Daejeon, 34134 Republic of Korea; 2 Department of Veterinary Internal Medicine, College of Veterinary Medicine, Seoul National University, Seoul, 08826 Republic of Korea; 3 Graduate School of New Drug Discovery and Development, Chungnam National University, Daejeon, 34134 Republic of Korea

**Keywords:** Dog, Orally disintegrating film formulation, Rectal administration, Sildenafil, Pulmonary hypertension

## Abstract

**Background:**

Sildenafil citrate, a highly selective phosphodiesterase type 5 inhibitor, is used to treat pulmonary hypertension (PH) in veterinary medicine. The objective of this study was to investigate pharmacokinetic profiles by oral administration of orally disintegrating film (ODF) and film coated tablet (FCT) formulations and rectal administration of ODF formulation in healthy dogs. Twelve healthy beagle dogs were administered four separate doses of sildenafil: FCT formulation 2 mg/kg orally, ODF formulation 2 mg/kg orally, ODF formulation 2 mg/kg rectally, and ODF formulation 10 mg/kg rectally. For 24 hours following administration, blood samples were collected and the plasma concentrations of sildenafil were assayed by liquid chromatography-tandem mass spectrometry.

**Results:**

There were no significant differences in all the pharmacokinetic parameters between FCT and ODF formulations when administrated orally. C_max_ at the time of rectal administration was lower when the same dose was given as that orally administered. No serious systemic adverse events (AEs) were observed.

**Conclusions:**

These findings suggest that sildenafil ODF formulation can be used as an alternative to FCT formulation in the treatment of canine PH patients; additionally, rectal administration of sildenafil ODF may be a beneficial treatment option for canine patients who are unable to receive medication orally.

## Background

PH is a complex syndrome characterized by a persistent, abnormal increase in pulmonary pressure resulting in life-threatening respiratory distress [1]. PH in dogs can be primary or secondary to other diseases, including heart and pulmonary disease [1, 2]. The most commonly reported presenting complaints in canine PH patients are exercise intolerance (45%), cough (30%), respiratory difficulty (28%), and syncope (23%) [3]. In veterinary medicine, sildenafil can decrease pulmonary arterial pressure and improve quality of life of canine patients with PH [4–7]. In particular, patients with PH who have respiratory distress are required to receive sildenafil. Sildenafil citrate (Viagra®) is a highly selective phosphodiesterase type 5 inhibitor that enhances nitric oxide mediated pulmonary vasodilatation [8]. Sildenafil is currently used only in oral formulations in veterinary medicine. However, attempting to administer oral medication to canine patients who are in respiratory distress or are receiving oxygen treatment may further exacerbate respiratory conditions or induce aspiration pneumonia. Thus, it is important to consider other forms of medication administration that do not interfere with respiration, such as rectal administration. No animal studies for these alternatives have been conducted so far.

In human medicine, there are two formulations of sildenafil: FCT and ODF. FCT should be administered with water, but ODF is easy to take without water due its effective solubility in the mouth. Owing to the presence of a larger surface area of this formulation and a highly vascularized oral or buccal mucosa, ODF provides rapid disintegration and dissolution in the oral cavity; a previous study show that the pharmacokinetics between FCT and ODF formulation do not differ significantly in healthy humans [9]. Due to these advantages, this formulation of drug could be much more suitable for an emergency patient with respiratory distress. Unfortunately, there are no studies that evaluate the pharmacokinetics of ODF formulation of sildenafil in veterinary medicine.

The first objective of this study was to compare the pharmacokinetic profiles of ODF formulation with those of a FCT formulation in healthy dogs when administered orally. The second objective was to investigate pharmacokinetic profiles to determine a dosage for rectal administration of ODF formulation of sildenafil that produced similar therapeutic effects to oral administration.

## Results

**Fig. 1:**
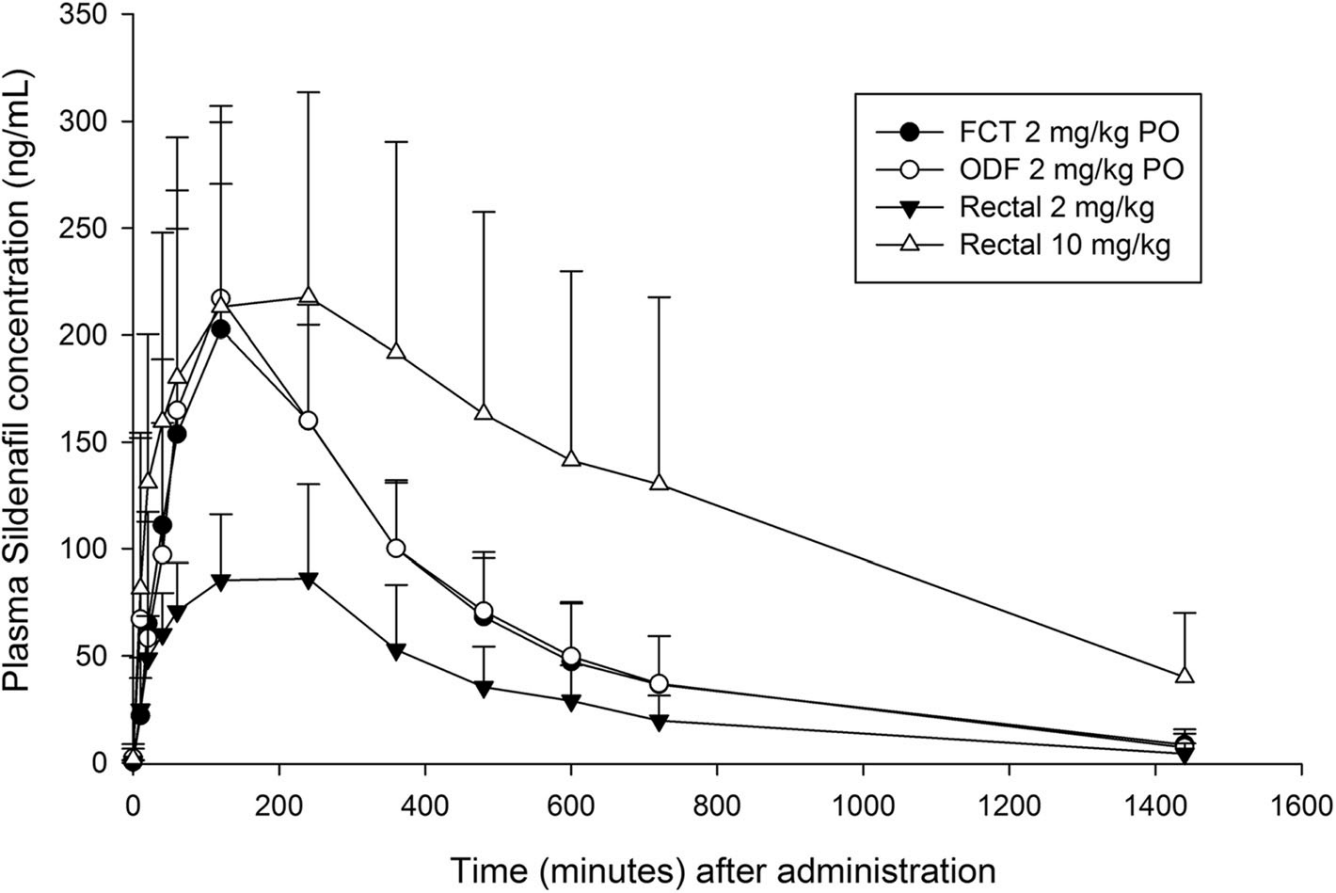
Mean plasma sildenafil concentrations for each arm. Plasma concentration-time profiles of sildenafil in beagle dogs after single-dose oral and rectal administration. FCT = Film-coated tablet PO; ODF = Orally disintegrating film PO

**Fig. 2:**
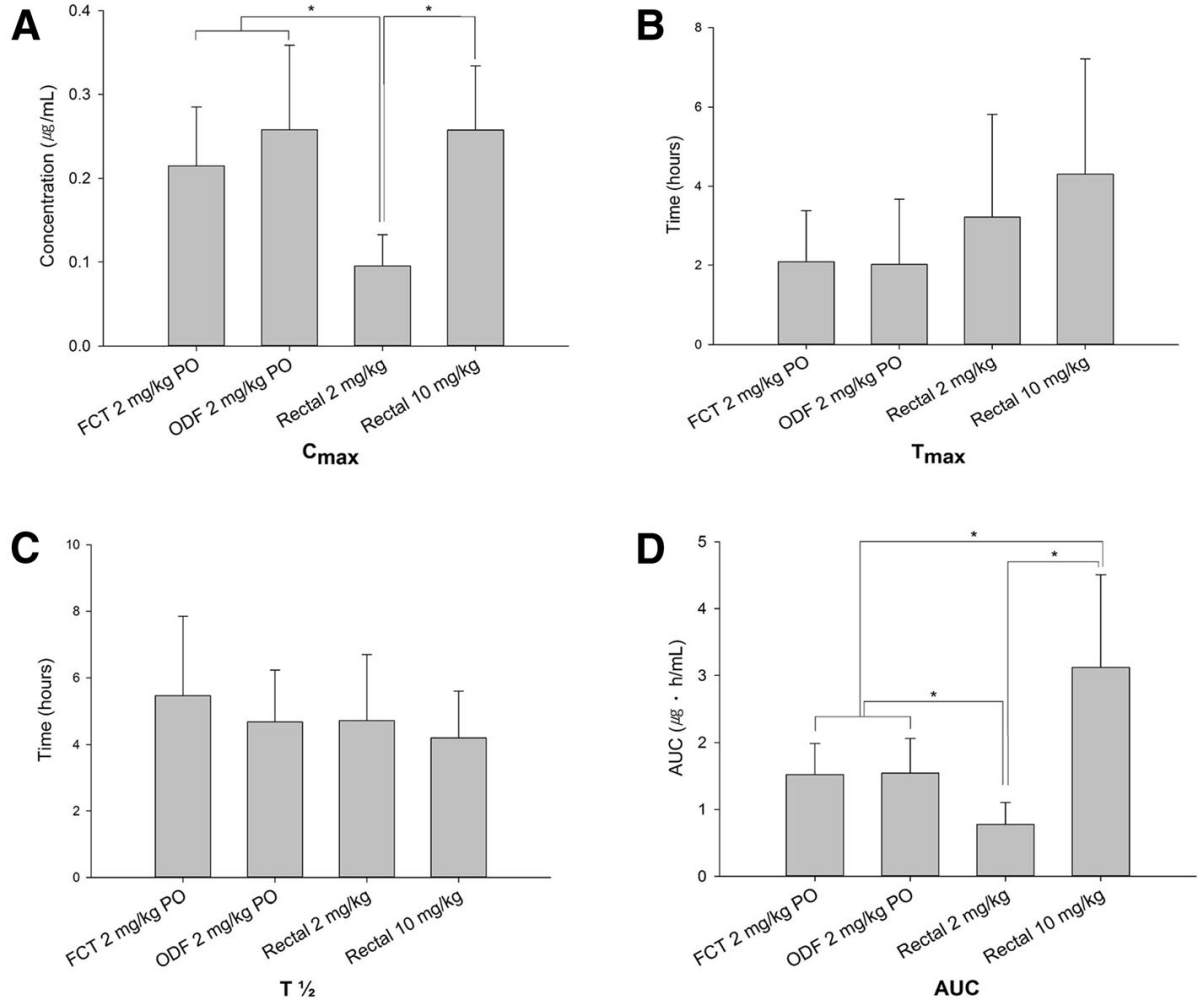
Comparison of pharmacokinetic parameters between four treatment arms (**a**, **b**, **c** and **d**). C_max_ = Maximum plasma concentration. T_max_ = Time at the maximum concentration. T_1/2_ = Elimination half-life. AUC_last_ = Area under the curve from time zero to time of last measurable concentration. FCT = film-coated tablet PO. ODF = orally disintegrating film PO. *Significantly different (*p* < 0.05)

The mean plasma drug concentrations of each formulation, route, and dosage are depicted in the Fig. 1. Comparisons of pharmacokinetic parameters between the four arms of treatment are presented in Table 1 and Fig. 2. There were no significant differences in pharmacokinetic parameters between the FCT and ODF formulations when administrated orally. There were increases in C_max_ and AUC_last_ between the rectal 2 mg/kg and 10 mg/kg administrations but the increases in values were not dose-proportional. Significant differences in C_max_ and AUC_last_ were observed between oral administrations and 2 mg/kg rectal administration. AUC_last_ was significantly greater after 10 mg/kg rectal administrations when compared to 2 mg/kg oral administrations. No significant differences were noted in the T_1/2_ and T_max_ among the four arms.

**Table 1:** Pharmacokinetic parameters of sildenafil in beagle dogs after administration of each arm

Pharmacokinetic parameters	FCT^a^ PO 2 mg/kg	ODF^b^ PO 2 mg/kg	Rectal^c^ 2 mg/kg	Rectal 10 mg/kg
C_max_ (μg/mL)	0.21 ± 0.07	0.25 ± 0.10	0.09 ± 0.04	0.26 ± 0.08
T_max_ (hours)	2.08 ± 1.30	1.88 ± 1.65	3.28 ± 2.49	4.30 ± 2.91
T_1/2_ (hours)	5.46 ± 2.39	4.62 ± 1.49	4.82 ± 1.91	4.20 ± 1.40
AUC_last_ (μg·h/mL)	1.52 ± 0.47	1.51 ± 0.51	0.77 ± 0.31	3.12 ± 1.39

*C*_*max*_ Maximum plasma concentration. *T*_*max*_ Time at the maximum concentration. *T*_*1/2*_ Elimination half-life. *AUC*_*last*_ Area under the curve from time zero to time of last measurable concentration

^a^FCT, Film-coated tablet, ^b^ODF, orally disintegrating film, ^c^Rectal, ODF rectal administration

No serious AEs, such as fatal or life-threatening AEs, or those requiring professional intervention, were observed during the study period. In three dogs, mild vomiting occurred 2 to 3 h after dosing (two in FCT 2 mg/kg and one in 2 mg/kg rectally). Soft stools were observed in five dogs (one in ODF 2 mg/kg, two in 2 mg/kg rectally, and two in 10 mg/kg rectally). In the subjects who received sildenafil 10 mg/kg rectally, soft stools occurred within 15 to 80 min after administration. Soft stools occurred in at least 2 h after administration in the other three dogs.

## Discussion

To our knowledge, this study is the first to evaluate the pharmacokinetic profiles of oral and rectal administration of sildenafil ODF formulation in dogs. This study demonstrates that the pharmacokinetic profiles after oral administration of the ODF formulation are very similar those of the FCT formulation in healthy dogs.

In canine patients with respiratory failure due to PH, sildenafil selectively acts on the pulmonary vascular smooth muscle, which reduces the risk of lowering systemic blood pressure [10, 11]. Therefore, it should be relatively safe to administer in an emergency. In human patients, ODF is administered in the form of one or two films. However, in this study, the films used in the ODF oral administration were cut into pieces as calculated by the body surface area of each subject. In dogs, the ODF dissolved on the tongue very quickly without water, just as it does in humans. This study incidentally revealed that ODF formulation of sildenafil is suitable for dogs even if it is administered in the form of cut pieces. Due to the ease of administration and accurate dosage, oral administration of sildenafil ODF could be an appropriate alternative for canine patients with PH who are not able to swallow any medication. This poses as an advantage since it broadens the prescription options available to veterinarians.

We performed rectal administration of sildenafil ODF formulation, which is rapidly absorbed in the mucous membranes [12]. Some studies have reported rectal administration of medication as an alternative route for dogs that are not able to receive medication orally because of seizure [13, 14]. However, previous studies demonstrated that the dosage for rectal administration should be adjusted because C_max_ and AUC_last_ (an indicator of bioavailability) after rectal dosing is relatively lower than that after oral administration. Previous studies have also shown that when the same dosage is given, the mean C_max_ is about 1.5 to 2.5 times higher when administered orally than when administered rectally [13, 15, 16]. This result demonstrates that rectal administration requires a higher dose than oral administration for obtaining similar plasma concentration. Based on previous research results, we performed a pharmacokinetic evaluation using a dose of sildenafil that was five times greater than that used in oral administration considering the possibility that sildenafil may leak out of the rectum. AUC_last_ after 10 mg/kg rectal administration was significantly higher than that after oral administration (*p* < 0.05). This result suggests that the dogs were overdosed with the 10 mg/kg rectal administration when compared to the 2 mg/kg PO treatments. Therefore, it is necessary to further investigate the dosage of rectally administered sildenafil that is needed to achieve the same effect produced from oral administration.

Although rectal administration had a tendency to delay the mean T_max_ more than oral administration, there were no significant differences in T_1/2_ and T_max_ in the four arms. As a result, regardless of the formulation or administration route of sildenafil, the timing of the clinical effect of the drug can be roughly estimated, which can be helpful for PH treatment in practice.

According to a previous report, the absorption of a drug decreases in the presence of feces [14]. In an effort to minimize the effects of feces on drug absorption in our study, sildenafil was administered rectally after fasting and administration of enemas. Although we fasted the dogs for 20 h, some dogs defecated after administration of the enema; this is because each dogs’ peristaltic movement time may vary [17]. Comparing the standard deviation values of C_max_ according to the route of administration, it can be concluded that consistent drug absorption occurred after rectal administration. However, it is difficult to perform an enema in canine patients with dyspnea in practice. Furthermore, other factors can affect the pharmacokinetics in rectal administration, including environmental pH, lipophilicity of the drug, and the site of drug deposition in the rectum [18–20]. Further research may be needed to consider these factors on rectal administration of sildenafil in canine patients.

According to the criteria for AEs, serious AEs were not observed [21]. Although six dogs showed mild vomiting (three events) or soft stool (five events), all dogs from the four arms of the study tolerated the experiment well. During the rectal administration, the vomiting and soft stools were suspected to be AEs of sildenafil given that they were observed after the enema for at least 15 min. It is reported that gastrointestinal upset is a possible side effect when sildenafil is administered [22].

This study has some limitations. First, the rectal dosing was administered in only two dosages. The rectal dosing equivalent to the effects 2 mg/kg of oral dosing was not known. Second, an active metabolite of sildenafil (N-desmethyl-sildenafil) was not tested. Sildenafil is metabolized to its active metabolite (N-desmethyl sildenafil), which accounts for about one-fifth of the drug’s activity [23]. Third, this study was conducted only with healthy intact male beagle dogs. Additional pharmacokinetic studies on canine patients with PH are needed. Fourthly, accurate bioavailability measurements using intravenous sildenafil were not conducted since intravenous sildenafil was not available.

## Conclusions

The pharmacokinetic profiles of oral administration were not significantly different between sildenafil FCT and ODF. Bioavailability with rectal administration was lower compared with oral administration of the same dose. All the dogs tolerated the experiment well without serious AEs. These results suggest that sildenafil ODF formulation can be used interchangeably with the FCT formulation and that rectal administration of ODF could be beneficial to canine PH patients. Additional studies would be needed to determine the appropriate rectal dose of sildenafil to achieve therapeutic levels similar to those obtained with oral administration.

## Methods

### Animals

Twelve intact male beagle dogs from a research colony at the College of Veterinary Medicine of Chungnam National University were included in the study. The animals were fed commercial dry food and had free access to water. The dogs were 4–7 years of age and weighed 9–13 kg. They each had normal physical and neurologic examinations; the complete blood counts and serum chemistry panels revealed no abnormalities, and the systemic systolic blood pressure, measured by Doppler method, was within normal range. This study obtained the approval of Institutional Animal Care and Use Committee (IACUC) at Chungnam National University (approval number, CNU-00749). After the study, 2 dogs were adopted as pets and the rest are waiting for adoption.

### Experimental design and sample collection

This was a randomized, open-label, single-dose, 4-way crossover study performed to evaluate sildenafil FCT and ODF with a washout period of 1 week. Before the administration of sildenafil, foods were withheld for over 20 h. Each dog received four separate doses of sildenafil in the following forms, dosages, and routes: (1) FCT formulation of sildenafil 2 mg/kg orally (FCT 2 mg/kg PO), (2) ODF formulation of sildenafil 2 mg/kg orally (ODF 2 mg/kg PO), (3) ODF formulation of sildenafil 2 mg/kg dissolved in distilled water (2 mg/ml) rectally (Rectal 2 mg/kg), and (4) ODF formulation of sildenafil 10 mg/kg dissolved in distilled water (3.3 mg/ml) rectally (Rectal 10 mg/kg).

FCT formulation of sildenafil (Viagra®) and ODF formulation of sildenafil (Viagra-L®) were purchased from Pfizer Korea Pharm. Co. (Seoul, South Korea). For FCT oral administration, sildenafil citrate was prepared in capsule form. For ODF oral administration, ODF formulation of sildenafil was cut according to the body weight of each subject. In preparation for rectal administration, ODF formulation of sildenafil was diluted in distilled water just before administration [13]. Given that the solubility of ODF is 3.5 mg/ml, the solution was diluted to a concentration lower than that. After administering an enema, this solution was administered rectally using a 10 mL or 30 mL syringe with a 9-cm flexible zonde [14]. The zonde was flushed with 1 mL of additional distilled water, and the anus was closed manually for 5 min to prevent early expulsion of the drug. Baseline blood collection was performed just before each administration. Eleven additional blood samples were collected at 10, 20, 40, 60, 120, 240, 360, 480, 600, 720, and 1440 min, respectively, after administration. All blood samples were collected from the jugular vein with a 23 g, 5 mL syringe. All of these were immediately drawn into heparinized tubes, and then the plasma was separated within 30 min and immediately frozen at − 80 °C. A small meal was provided to the dog after blood collection, which was performed 4 h after sildenafil administration.

Throughout the study, the dogs were observed for AEs for 24 h after each drug administration. In order to evaluate AE, we continuously monitored the clinical symptoms and performed physical examinations. Systemic systolic blood pressure was monitored by Doppler method. Four doses had been missed due to animal hyperactivity and technical error (one in ODF orally, one in 2 mg/kg rectally, two in 10 mg/kg rectally). Data from those four missed doses were excluded.

### Measurement of plasma sildenafil concentration

The plasma concentration of sildenafil was determined by liquid chromatography–tandem mass spectrometry. For the high-performance liquid chromatography (HPLC) separation and the tandem mass spectrometry detection, plasma samples were pretreated using the following method. An aliquot (50 μL) of internal standard solution (carbamazepine 10 ng/mL in acetonitrile) was added to an aliquot (50 μL) of plasma. Thereafter, 400 μL of acetonitrile was added to the samples to induce the precipitation of plasma proteins. The resulting mixture was mixed vigorously for 10 min and centrifuged at 13,500 rpm for 10 min. An aliquot (5 μL) of supernatant was directly injected into the LC-MS/MS system.

The separation by chromatography was performed with reverse phase column (Agilent ZORBAX C18, 3 μm, 2.1 × 50 mm) equipped with guard column (Agilent ZORBAX C8, 5 μm, 2.1 × 12.5 mm) and Agilent HPLC system (Agilent Technologies, Santa Clara, CA, USA). Detection was conducted by triple quadrupole tandem mass spectrometer system (API 4000, Applied Biosystems/MDS SCIEX, Foster City, CA, USA). The molecular ion fragmentation of sildenafil and carbamazepine was performed under the condition of collision energy at 53 V and 29 V, respectively, by collision-activated dissociation with nitrogen as the collision gas. A multiple reaction monitoring mode was used for quantification at m/z 475.2 to 283.2 for sildenafil and m/z 237.2 to 194.2 for carbamazepine. The peak area for all components were automatically integrated using Analyst software version 1.5.1 (Applied Biosystems/MDS SCIEX). The quantifiable range of the plasma samples was 0.001 to 10 μg/mL. The retention times of sildenafil and carbamazepine were 4.11 min and 4.26 min, respectively.

### Analysis of the data

To estimate the pharmacokinetic parameters of sildenafil, the plasma concentration for each beagle dog was analyzed by non-compartmental model analysis with non-linear regression using WinNonlin 4.1 software package (Pharsight, Cary, NC). The elimination rate constant (ke) was determined by linear regression of the log-linear portion of the terminal phase. The terminal elimination half-life (T_1/2_) was calculated by dividing the natural logarithm of 2 (0.693) under each ke value. The area under the sildenafil concentration-versus-time curve, from time zero to the time of the last quantifiable concentration (AUC_last_), was calculated using the linear trapezoidal rule and the standard area extrapolation method from WinNonlin 4.1. The C_max_ and T_max_ were directly compiled from the concentration-time curves. One-way ANOVA was used to evaluate differences in the pharmacokinetic parameters among treatment arms. If there were any differences, Duncan’s test was used to determine if these differences were significant or not.

## Abbreviations

AE: Adverse event
AUC_last_: Area under the curve from time zero to time of last measurable concentration
C_max_: Peak plasma concentration
FCT: Film-coated tablet
HPLC: High-performance liquid chromatography
ke: Elimination rate constant
LC-MS/MS: Liquid chromatography–tandem mass spectrometry
ODF: Orally disintegrating film
PH: Pulmonary hypertension
T_1/2_: Half-life
T_max_: Time to reach the peak plasma concentration
